# Tomographic late evaluation of xenogeneic bone grafts in sockets of impacted third molars

**DOI:** 10.1590/1678-7757-2017-0396

**Published:** 2018-07-06

**Authors:** Osny FERREIRA JÚNIOR, Etiene Andrade MUNHOZ, Jéssica de Fátima SEGANTIN, Eduardo Sanches GONÇALES, Paulo Sérgio Perri de CARVALHO

**Affiliations:** 1 Universidade de São Paulo, Faculdade de Odontologia de Bauru, Departamento de Cirurgia, Estomatologia, Patologia e Radiologia, Bauru, São Paulo, Brasil.; 2 Universidade Federal de Santa Catarina, Centro de Ciências da Saúde, Departamento de Estomatologia, Florianópolis, Santa Catarina, Brasil.

**Keywords:** Tomography, Heterografts, Third molar, Bone regeneration

## Abstract

It is necessary to preserve height and thickness of the alveolar bone to facilitate rehabilitation with osteointegratable implants or simply to maintain bone integrity after extraction. Biomaterials associated with resorbable or non-resorbable membranes, when placed in the region of the socket, may contribute to avoid this unwanted reabsorption.

**Objective:**

The objective of this study was to evaluate the distance of the crest of alveolar ridge to the cementoenamel junction (CEJ) of the lower second molars and the bone density of the third molar socket filled with Gen-Tech^®^, 5 years after an exodontia using cone beam computed tomography (CBCT) to visualize the central region of the sockets, without overlapping of the buccal and lingual cortical bones.

**Material and Methods:**

A total of 12 individuals from an initial group of 39 patients submitted to extraction of the unruptured lower third molars and grafting of an association of inorganic bovine bone matrix, organic bovine bone matrix, collagen and bone morphogenetic proteins (BMP) (Gen-Tech^®^) on one side and the contralateral sockets filled only by clot, returned to control after 5 years, and were submitted to CBCT. The distance from the crest of alveolar bone to the CEJ and the bone density (BD) were measured using the i-CAT Vision Software.

**Results:**

The results showed that the distance from the crest of alveolar bone to the CEJ in the control group was similar to that observed before the exodontia; in the experimental group, this distance was smaller. Considering the BD measurement, a significantly higher density was observed in the experimental group (p<0.05).

**Conclusion:**

Part of the biomaterial was not absorbed and allowed the stability of the evaluated parameters after 5 years, being able to be used as a bone substitute in the socket.

## Introduction

Extraction of the impacted third molars is a procedure commonly performed by maxillofacial surgeons to avoid pericoronitis, odontogenic cysts and tumors, caries, reabsorption and periodontal problems of the adjacent teeth.^12^ ^,^ ^18^ ^,^ ^21^ The filling of the sockets with clot alone is, in most cases, sufficient to obtain a good periodontal condition of the second molars without the need for additional procedures.^4^

However, factors such as infection and maintenance of the clot may postpone the bone repair of this region.^5^ Some studies claim that the third molar removal can result in periodontal bone defects on the distal surface of the adjacent second molar. To maintain bone height, thickness and quality in this region, biomaterials used as bone substitutes or associated with autogenous bone have been used to fill the socket of the third molars.^13^ ^,^ ^14^ ^,^ ^21^ ^,^ ^23^

In other regions of the mouth, the preservation of the alveolar bone after extraction becomes even more important. In a recent systematic review and meta-analysis, Troiano et al.^4^ (2017) emphasized the importance of maintaining the height and thickness of the alveolar ridge, comparing an experimental group of sockets filled with bone substitutes, allogeneic and xenogeneic, covered by resorbable membranes, with a control group of sockets with spontaneous healing, i.e., filled with clot only. The results showed lower resorption of both thickness and height of the alveolar ridge for the experimental group.

To avoid bone loss, especially of the buccal plate of anterior teeth, should be a concern when rehabilitating aesthetic areas. This loss is directly related to the presence or absence of the buccal plate and its thickness. If the buccal plate is 2 mm thick or thicker, it is not necessary to fill this socket, either using biomaterials or autogenous bone. This concern is lower when the area to be rehabilitated with implants is more posterior, because, in general, the thickness of the buccal plate is larger and there is no aesthetic involvement.^9^

There is an immense variety of these biomaterials constantly available, whose main function is to serve as a scaffold for the clot, aiding in the maintenance of the post-extracted alveolar bone. To achieve a satisfactory result the main properties of these materials are biocompatibility and osteoconduction, and they may or may not be associated with resorbable or non-resorbable membranes, which prevent the migration of connective tissue cells into the socket under repair.^1^ ^,^ ^24^

To evaluate biomaterials in the dental socket, the third molars provide a widely used model of study, either by the large number of patients requiring this type of surgery or by the ease of finding third molars in a symmetrical position on the right and left side of the same patient, allowing split-mouth studies.^21^

Some studies have used both conventional intraoral and panoramic radiographs.^2^ ^,^ ^7^ ^,^ ^8^ ^,^ ^10^ ^,^ ^11^ ^,^ ^15^ Others have compared the images obtained in panoramic radiographs and tomographies evaluating biomaterials, proximity of the third molar to the lower alveolar nerve and external root resorption.^3^ ^,^ ^6^ ^,^ ^18^ ^,^ ^20^ ^,^ ^22^ The results confirm that cone beam computed tomography (CBCT) has better image quality, better measurement accuracy, less distortion and, mainly, allows the visualization of the region of interest (ROI) without overlapping other structures.^1^ ^,^ ^6^ ^,^ ^16^ ^,^ ^17^ ^-^ ^20^ ^,^ ^25^

Our study analyzed the distance from the crest of alveolar ridge to the CEJ at the surface of distal second molars and the bone density of the lower third molar socket filled with an association of inorganic bovine bone matrix, organic bovine bone matrix, collagen and bone morphogenetic proteins (BMP) (Gen-Tech^®^) and compared with the clonally filled contralateral sockets using CBCT.

## Material and methods

CBCTs were analyzed from 12 individuals belonging to the set of 39 patients from the study of Munhoz et al.^15^ (2006), who returned for evaluation after 5 years.

The inclusion criteria to select patients undergoing surgery in that study were: age between 15 and 25 years; presence of impacted lower third molars in a symmetrical position according to the classification of Winter and Pell & Gregory; indication for extraction; and absence of systemic disease. Pregnant women and smoking patients were excluded. All patients were instructed about the procedures, in addition to receiving and signing an informed consent form. The research was approved by the Ethics Committee of the Bauru School of Dentistry, University of São Paulo.

**Figure 1:**
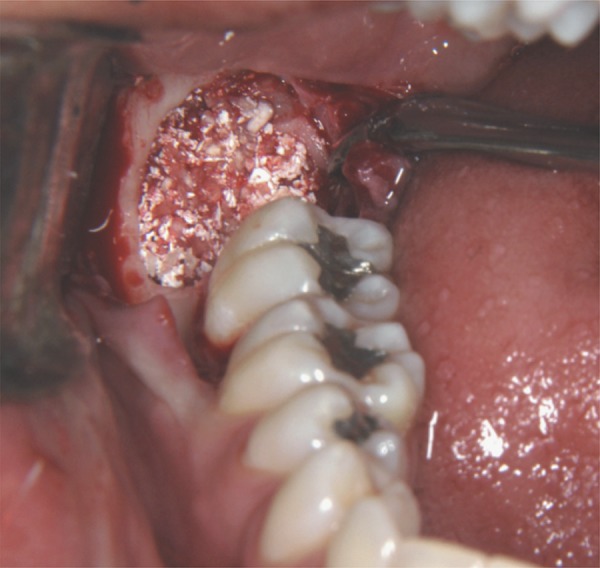
Sockets filled with Gen-Tech® in study conducted by Munhoz, et al.^15^ (2006)

Each patient had one socket in the control group and the other in the experimental group, randomly selected, setting up a randomized study. In the control group, the socket was filled only by clot. In the experimental group, the socket was filled with Gen-Tech^®^ (Baumer SA., Mogi Mirim, SP, Brazil), a biomaterial composed of bovine bone organic matrix + inorganic bovine bone + collagen + BMP, previously hydrated with saline solution (Figure 1). A freeze-dried bovine bone cortical membrane (Gen-Derm^®^) (Baumer SA., Mogi Mirim, SP, Brazil) was then placed and sutured.

The i-CAT Classic^®^ device (Imaging Sciences International, Hatfield, Pennsylvania, USA) was used to perform the tomographic exams with the following protocol: field of view (FOV) of 6 cm, voxel size of 0.3 mm and scan time of 20 s. On the “implant screen” screen of the i-CAT Vision^®^ software, adjustments were made for brightness, contrast and points used to determine the panoramic reformatting plan. The thickness of this plan (15 to 3 mm) was also altered to obtain a reformatting that went right through the center of the third molar socket.

**Figure 2:**
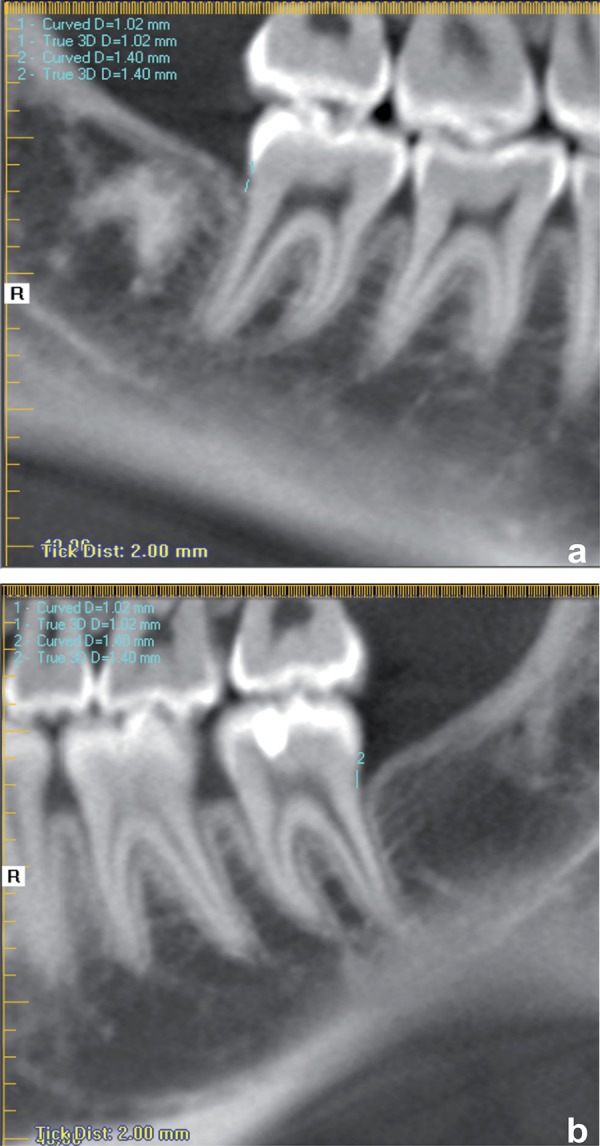
Measurement from the crest of alveolar bone to the cementoenamel junction (CEJ) filled by biomaterial (a) and by clot (b)

**Figure 3:**
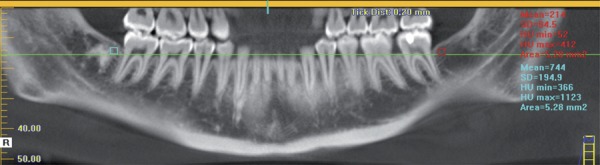
Measurement of bone density in the control and experimental groups

Measurements of distance from the crest of alveolar bone to the CEJ in the distal second molars were made using the distance tool, drawing a line from the most coronal point of the bone crest to the most apical point of the enamel (Figures 2a and 2b). Measurements of bone density were made using the “HU statistics” tool with which a square with 5.28 mm_2_ of area was drawn, located 5 mm from the distal face of the second molar and 1 mm above a horizontal line determined by 2 points located in the mesial bone crest of the lower second molars (Figure 3).

All measurements were repeated after 15 days by the same examiner. The concordance index was calculated by the Dahlberg’s Error test. The results were analyzed by the paired t-test. A significance level of 5% was adopted for all tests.

## Results

**Figure 4:**
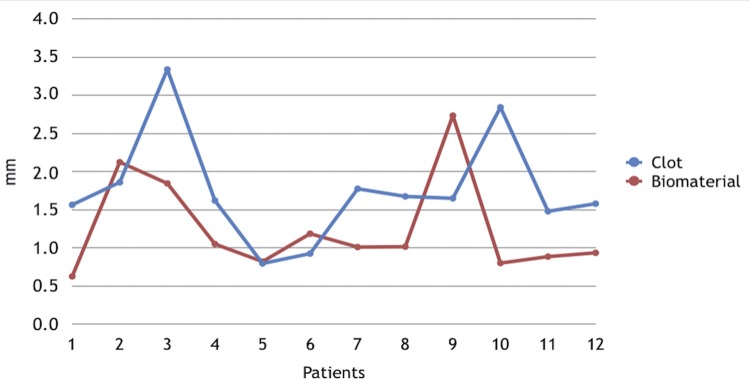
Measurement, in mm, for the distance between the crest of alveolar bone and the cementoenamel junction (CEJ) in the distal second molars in each patient

**Figure 5:**
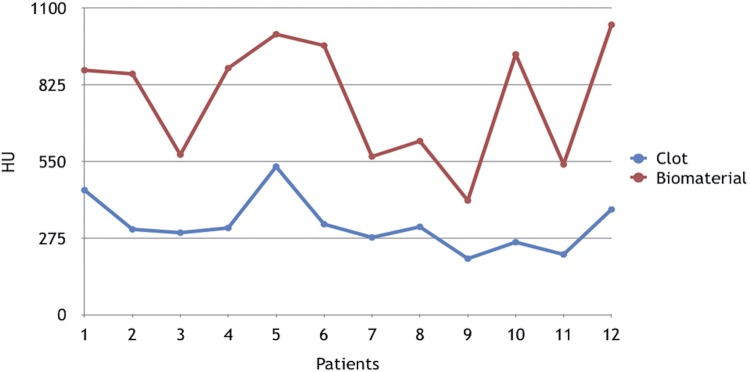
Measurement, in HU, of the bone density of clot-filled socket and biomaterial in each patient

Tables 1 and 2 and Figures 4 and 5 show the results obtained regarding the measurement of the distance from the crest of alveolar bone to the cementoenamel junction in the distal surface of the second molars and the bone density measured according to the methodology described previously.

**Table 1:** Measurement (mm) of the distance from the crest of alveolar bone to the cementoenamel junction (CEJ) in the distal second molars

	clot		biomaterial		t	p
	mean	SD	mean	SD		
distance	1.774	0.736	1.263	0.646	2.114325	0.058139 ns

ns= no statistically significant difference

**Table 2:** Measurement (HU) of bone density of the third molar socket

	clot		biomaterial		t	p
	mean	SD	mean	SD		
density	322.16	93.10	774.16	215.59	-9.34725	0.000001 sd

sd= statistically significant difference

As shown in Table 1, the distance from the crest of alveolar bone to the CEJ on the distal surface of the second molars after extraction of the third molars was, on average, 1.774 mm, with a standard deviation of 0.736 mm in the control group and 1.263 mm, with standard deviation of 0.646 mm in the experimental group. There was no statistically significant difference between groups (p=0.058139).

Averages of bone densities were 322.166 HU for the control group and 774.166 HU for the experimental group, showing statistical difference between groups (p=0.000001) (Table 2).

## Discussion

In this study, measurements of the distance from the crest of alveolar bone to the CEJ and the density measurements, evaluated in the tomographic examinations, showed good intra-examiner agreement, evaluated by the paired t-test, with p=0.661 for distance measurements and p=0.153 for the density measurements.

Although no statistical difference was observed between groups, our study differs from others in relation to the time of postoperative control, since the periods are shorter in most studies.^3^ ^,^ ^6^ ^,^ ^20^

Thus, when analyzing the evolution of the measurements of distance from the crest of alveolar bone to the CEJ, in the same patients over time, considering the initial measurements of the study of Munhoz, et al.^15^ (2006), we can verify that, after 5 years, in the clot-filled group, this parameter returned to preoperative levels, while in the group filled with Gen-Tech^®^, this distance was lower. The distance reduction of the crest of alveolar bone to the CEJ also occurred compared to the 2-year postoperative period.^14^

A similar study was performed by Singh, et al.^21^ (2015), comparing the efficacy of an alloplastic material compared with an absorbable gelatin sponge in the prevention of distal periodontal defects to the second lower molar after surgical removal of impacted lower third molars. This study also observed an increase in the level of alveolar bone and improvement of periodontal disease in the group with hydroxyapatite and collagen graft compared to the sponge group (Abgel, Shri Gopal Krishna Labs Pvt. Ltd., Mumbai, India). Regarding the density measurements, the mean of the experimental group remained higher than that of the control group after 5 years, evidencing the permanence of the biomaterial. Other studies corroborate the late reabsorption of this type of material.^13^ ^,^ ^16^ ^,^ ^21^

One point to be highlighted in our study is the use of CBCT to evaluate bone repair in the socket of third molars with or without the use of biomaterials. This is a tool more accurate than that used in the studies of Munhoz, et al.^15^ (2006), who used panoramic and periapical radiographs and the same group of individuals. In addition, CBCT allows measurements to be performed without distortion or overlap of structures.

Oenning, et al.^18^ (2014) compared the panoramic radiograph and CBCT to evaluate the external root resorption (ERR) of the second molars associated with impacted third molars. In this study, Oenning, et al.^18^ (2014) evaluated 66 subjects and significantly obtained more cases of ERR (p<0.0001) diagnosed with CBCT (n=43 or 22.88%) when compared to panoramic radiographs (n=10 or 5.31%), confirming the greater precision of CBCT and justifying its use in this type of evaluation.

Finally, this evaluation showed that the biomaterial used did not undergo complete resorption in the analyzed period, contrary to what is expected of a bone substitute, which leads to the need for studies to analyze the integration of implants into biomaterial filled cells.^13^ ^,^ ^14^ ^,^ ^18^ ^,^ ^21^ ^,^ ^23^

## Conclusions

1- The distance from the crest of alveolar bone to the cementoenamel junction did not show a significant difference between the groups, even though smaller distances were observed in the group filled with the biomaterial;

2- There is a significant difference between the groups considering the bone density;

3- The biomaterial allowed the stability of the evaluated parameters after 5 years, and can be used as a bone substitute in the socket;

4- There is permanence of unabsorbed biomaterial even after 5 years.
